# Behavioral History of Withdrawal Influences Regulation of Cocaine Seeking by Glutamate Re-Uptake

**DOI:** 10.1371/journal.pone.0163784

**Published:** 2016-09-29

**Authors:** Luyi Zhou, Haley Andersen, Adrian C. Arreola, Jill R. Turner, Pavel I. Ortinski

**Affiliations:** 1 Department of Pharmacology, Physiology, and Neuroscience, University of South Carolina School of Medicine, Columbia, SC, 29208, United States of America; 2 South Carolina College of Pharmacy, Department of Drug Discovery and Biomedical Sciences, University of South Carolina, Columbia, SC, 29208, United States of America; 3 Center for Neurobiology and Behavior, Department of Psychiatry, Perelman School of Medicine at the University of Pennsylvania, Philadelphia, PA, 19104, United States of America; Radboud University Medical Centre, NETHERLANDS

## Abstract

Withdrawal from cocaine regulates expression of distinct glutamate re-uptake transporters in the nucleus accumbens (NAc). In this study, we examined the cumulative effect of glutamate re-uptake by multiple excitatory amino acid transporters (EAATs) on drug-seeking at two different stages of withdrawal from self-administered cocaine. Rats were trained on fixed ratio 1 (FR1), progressing to FR5 schedule of reinforcement. After one day of withdrawal, microinfusion of a broad non-transportable EAAT antagonist, DL-threo-beta-benzyloxyaspartate (DL-TBOA), into the NAc shell dose-dependently attenuated self-administration of cocaine. Sucrose self-administration was not affected by DL-TBOA, indicating an effect specific to reinforcing properties of cocaine. The attenuating effect on cocaine seeking was not due to suppression of locomotor response, as DL-TBOA was found to transiently increase spontaneous locomotor activity. Previous studies have established a role for EAAT2-mediated re-uptake on reinstatement of cocaine seeking following extended withdrawal and extinction training. We found that blockade of NAc shell EAATs did not affect cocaine-primed reinstatement of cocaine seeking. These results indicate that behavioral history of withdrawal influences the effect of re-uptake mediated glutamate clearance on cocaine seeking. Dynamic regulation of glutamate availability by re-uptake mechanisms may impact other glutamate signaling pathways to account for such differences.

## Introduction

Repeated cocaine has been linked to changes in glutamate signaling in the NAc that may contribute to both maintenance of cocaine self-administration and reinstatement of cocaine seeking following extinction training [1,2,3,4,5]. Availability of glutamate, a critical determinant of neuronal signaling strength, is regulated by behavioral history of withdrawal from cocaine. After both short (1–2 days) and long (10–30 days) withdrawal from repeated cocaine, reduced basal NAc glutamate levels are observed [1,6,7]. Following long withdrawal combined with extinction training, glutamate has been observed to recover to baseline levels [8] or remain suppressed [9]. Dynamic regulation of extracellular NAc glutamate levels has been attributed to various sources, most prominently to extracellular glutamate clearance by distinct excitatory amino-acid transporters, EAAT1-3, and to non-vesicular release of glutamate through the cysteine-glutamate exchanger, xCT1. Long periods of withdrawal or extinction training have been associated with reduced activity of EAAT2 (also known as GLT-1) and xCT1 [10,11,12]. In contrast, following short withdrawal, elevated protein expression of EAAT1 and EAAT3, but no change or a slight decrease in EAAT2, has been observed in the NAc shell [10,13]. Combined with increased EAAT binding of glutamate [8] and reduced NAc glutamate levels [1,7,9], these findings support a net increase in glutamate re-uptake in early withdrawal.

Behavioral sensitivity to pharmacological treatments at different stages of cocaine experience also reflects involvement of distinct molecular substrates regulating glutamate availability. Following short withdrawal intervals, administration of ceftriaxone, a beta lactam antibiotic that increases EAAT2 expression, failed to affect cocaine taking and seeking, suggesting that EAAT2 activity is not behaviorally relevant at this stage [14]. However, after a long withdrawal from cocaine (with or without extinction), ceftriaxone attenuated reinstatement of cocaine seeking and reduced cocaine-induced locomotor sensitization [11,14,15], suggesting that decreases in glutamate availability in late withdrawal have suppressive effects on cocaine seeking. Paradoxically, N-acetyl-cysteine, administered to increase xCT1-mediated glutamate efflux was also found to suppress locomotor sensitization and reinstatement of cocaine seeking [6,12,16]. In an apparent solution to this discrepancy, the effects of N-acetyl-cysteine on reinstatement were recently reported to rely on up-regulation of EAAT2-mediated transport, rather than xCT1 activity [17], further highlighting the importance of glutamate re-uptake mechanisms in behavioral effects of cocaine.

Although glutamate re-uptake by EAAT2 plays a dominant role in clearing extracellular glutamate [18], other EAATs have been argued to regulate both neuronal function [19,20] and cocaine-associated behaviors [21]. However, cumulative effect of re-uptake by all EAATs on behavior is not known. We have recently demonstrated that short withdrawal from limited (2 hr) access cocaine self-administration is associated with increased EAAT activity that offsets the impact of altered NMDA receptor distribution in the NAc shell [13]. In this study, we wished to investigate whether up-regulation of NAc shell glutamate re-uptake after short withdrawal from cocaine self-administration may contribute to cocaine-related phenotypes. In a limited-access access paradigm, thought to model human recreational drug use [22], we administered a broad antagonist of EAATs, DL-TBOA, into the NAc shell and examined its effects on maintenance of cocaine self-administration (short withdrawal) and on cocaine-primed reinstatement of cocaine-seeking (long withdrawal with extinction).

## Materials and Methods

### Animals and housing

Male Sprague Dawley rats (*Rattus norvegicus*), weighing between 250–320 grams, were obtained from Taconic Laboratories (Germantown, NY, USA). Subjects were individually housed in a colony room with *ad libitum* food and water access and were maintained on a 12-h/12-h light dark cycle, with lights on at 0700 hours. All experimental procedures were followed in accordance with the University of Pennsylvania School of Medicine and University of South Carolina School of Medicine Institutional Animal Care and Use Committees.

### Drugs

Cocaine hydrochloride was a gift from the National Institute on Drug Abuse (Rockville, MD, USA). DL-threo-beta-benzyloxyaspartate (DL-TBOA) was obtained from Tocris Bioscience (San Diego, CA). All drugs were dissolved in bacteriostatic 0.9% saline.

### Surgery

**Fig 1:**
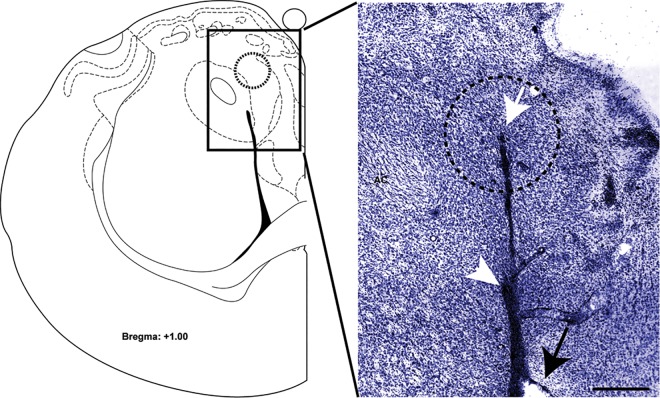
Cannulae placement verification. *Left*, A schematic of the brain section from Paxinos and Watson [40]. *Right*, A representative photomicrograph of cresyl violet-stained brain slice (75 μm-thick) used to confirm cannulae placements in the NAc shell. Dotted circle (1 mm diameter) represents the maximal diffusion area for a 0.5 μl solution volume [41]. Black arrow–tip of the guide cannula. White arrow–tip of the microinjector. White arrowhead–lateral ventricle. AC–anterior commissure. Scale bar, 0.5 mm.

After seven days of habituation to handling and home cage acclimation, rats were anesthetized with 80mg/kg ketamine and 12mg/kg xylazine (Sigma Aldrich/RBI, St. Louis, MO, USA). Then, an indwelling silicone catheter (CamCaths; Cambridge, UK) was inserted into the right jugular vein and sutured in place. The catheter was routed to a mesh mount and fixed subcutaneously above the scapula. Subsequently, animals were placed in a stereotaxic instrument (Kopf Instruments, Tujunga, CA, USA), and guide cannulae targeting the NAc shell were positioned using the following stereotaxic coordinates (in mm from bregma): A/P: +1, M/L +/- 1, D/V: -5. Guide cannulaes were fixed to the skull with dental acrylic. Stainless steel obturators were placed inside the cannulae to prevent occlusions. Cannulae placements were confirmed by histological analyses (Fig 1). Jugular catheters were flushed daily with 0.3 ml of a solution of the antibiotic Timentin (0.93 mg/ml; Fisher, Pittsburgh, PA, USA) dissolved in heparinized 0.9% saline (Butler Schein, Dublin, OH, USA) to prevent infection and maintain patency.

### Cocaine self-administration

One week after recovery from surgery, rats underwent daily two-hour cocaine self-administration sessions. Self-administration experiments were conducted in ventilated, sound attenuating operant chambers, equipped with a house light, active and inactive response levers, and an automated syringe pump for intravenous drug delivery (Med-Associates Inc., East Fairfield, VT, USA). All subjects began on a fixed-ratio one (FR1) schedule of reinforcement, in which each active lever press resulted in a 5-s intravenous cocaine infusion (0.25 mg/59 μl), followed by a 20-s timeout period during which the house light was turned off. Presses on the inactive lever had no consequences. No cue lights (other than the house light) were associated with the drug infusions. Once stable responding was achieved under the FR1 schedule (defined as 20 or more infusions over two consecutive sessions), the rats progressed to an FR5 schedule, in which five active lever presses were necessary to obtain a single infusion of cocaine. Overall, the rats were maintained on FR1 for 2–6 days, and on FR5 for 6–17 days.

### Extinction and reinstatement of cocaine seeking

Following self-administration and between reinstatement tests, rats underwent daily two-hour extinction sessions. During extinction sessions, cocaine was replaced with 0.9% bacteriostatic saline. Extinction was continued until active lever responding on an FR5 schedule was less than 15% of the average of the last 3 days of cocaine self-administration for two consecutive sessions. To induce reinstatement, animals received a priming injection of cocaine (10mg/kg; i.p.) immediately before the start of the self-administration session run under extinction conditions (FR5/saline infusions).

### Sucrose self-administration

Sucrose self-administration followed the same procedure as cocaine self-administration, with the following exceptions. First, sucrose animals only received NAc cannula implantation. Second, instead of cocaine infusions, rats received sucrose pellets (45mg, Noyes pellets, Fisher Scientific, Pittsburg, PA) as reinforcement. Last, rats were food restricted to maintain 85% of *ad lib* body weight.

### Experiment 1: Effect of DL-TBOA on maintenance of cocaine self-administration

To test the effect of DL-TBOA on maintenance of cocaine self-administration, 22 rats were first trained on FR1 until they met the criteria of >20 infusions over two consecutive sessions and then on FR5 schedule of reinforcement. Once they reached a stable baseline of cocaine self-administration defined as less than 20% variability on active lever presses in the last 5 self-administration sessions, they underwent a test cocaine self-administration session. On the test day, animals received a bilateral microinjection of vehicle (0.9% NaCl) or DL-TBOA (3 mM or 5 mM) into the NAc shell through microinjectors extending 2 mm below tips of the guide cannulae (0.5 μl/side over a 2 minute period). Microinjectors were then left in place for one additional minute to allow diffusion of the drug. Test self-administration sessions were initiated 10 minutes following the drug infusion.

### Experiment 2: Effect of DL-TBOA on cocaine-primed reinstatement

To examine the effect of DL-TBOA on reinstatement of cocaine seeking, a separate cohort of 7 animals was trained up to the FR5 schedule of reinforcement until they reached a stable baseline response, then underwent daily extinction sessions. All animals reached the extinction criterion before reinstatement tests. They then received a bilateral infusion of vehicle or DL-TBOA (5 mM, based on results from experiment 1) into the NAc shell 10 min prior to cocaine-primed reinstatement of cocaine seeking. Vehicle and DL-TBOA infusions were counterbalanced across animals over two reinstatement sessions. Separate extinction sessions were run between the two reinstatement sessions until extinction criteria were reached.

### Experiment 3: Effect of DL-TBOA on maintenance of sucrose self-administration

To test the effect of DL-TBOA on naturally rewarding stimuli, a group of 6 animals was trained to self-administer sucrose pellets on FR1 for 5 days and then on FR5 for the rest of the experiment. All animals reached a stable performance of <20% variability in active lever presses over the last five self-administration sessions both before the first test and between the two tests. On test day, rats received vehicle or DL-TBOA (5 mM) infusion into the NAc shell 10 min before the start of the test session. Vehicle and DL-TBOA infusions were counterbalanced across animals over two reinstatement sessions.

### Experiment 4: Effect of DL-TBOA on locomotor activity

Locomotor experiments were conducted in 16’×16’× 14.75’ square shaped open-field testing chambers, equipped with photobeam sensors, enclosed by four transparent plexiglass walls (San Diego Instruments, San Diego, CA, USA). Ten naïve animals that had not been exposed to cocaine were used for these experiments. Animals were first habituated to the locomotor testing chambers, and basal locomotor activity was recorded for 30 min. They were then removed from the chambers, given intracranial infusions of vehicle or DL-TBOA (5 mM) into the NAc shell and placed back into the locomotor chambers. Locomotor activity was then measured for the following 60 minutes, and quantified as total distance traveled.

### Data analysis

To evaluate the effect of DL-TBOA on maintenance of cocaine self-administration, one-way analyses of variance (ANOVA) followed by Bonferroni’s post-hoc tests were used to analyze the active/inactive lever presses and cocaine infusions. Paired t-tests were used for the analyses of DL-TBOA effect on reinstatement of cocaine seeking and the active/inactive lever responding and pellets earned during the maintenance phase of sucrose self-administration. Two-way repeated measures ANOVA followed by Bonferroni’s post-hoc test was used to analyze distance traveled during locomotor activity measurement. All data are presented as the mean±SEM with α set at p<0.05.

## Results

### Experiment 1: DL-TBOA dose-dependently decreases cocaine self-administration in early withdrawal

**Fig 2:**
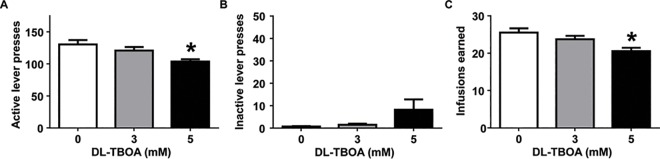
Microinjection of DL-TBOA into the NAc shell dose-dependently attenuated cocaine self-administration. A),B) Total number of responses (mean ± SEM) on the active and inactive levers following microinfusion of vehicle (n = 11), 3 mM (n = 4), and 5 mM (n = 7) of DL-TBOA into the NAc shell bilaterally (Bonferroni, *p<0.05 relative to vehicle controls). C) Total number of cocaine infusions (mean±SEM) earned during the two-hour self-administration sessions (Bonferroni, *p<0.05 relative to vehicle controls).

We first examined the effect of blocking glutamate reuptake in the NAc shell on cocaine self-administration following a 24-hour withdrawal. Microinfusion of DL-TBOA into the NAc shell attenuated active lever responding in a dose-dependent manner. One-way ANOVAs indicated significant group differences in active lever responding (F_(2,19)_ = 4.96, p<0.05; Fig 2A) and cocaine infusions (F_(2,19)_ = 5.49, p<0.05; Fig 2C). Post-hoc analyses indicated that 5 mM DL-TBOA, but not 3 mM DL-TBOA, significantly reduced both active lever presses and cocaine infusions. However, DL-TBOA did not significantly affect inactive lever presses (F_(2,19)_ = 2.83, p = 0.08; Fig 2B). These results indicate that activity of glutamate re-uptake transporters contributes to maintenance of cocaine seeking during early withdrawal from cocaine self-administration.

### Experiment 2: DL-TBOA has no effect on reinstatement of cocaine seeking after extinction training

**Fig 3:**
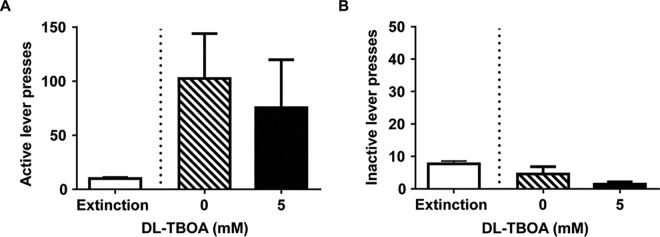
Microinjection of DL-TBOA did not affect cocaine priming-induced reinstatement of cocaine seeking. A), B) Total number of responses (mean ± SEM) on the active and inactive levers during extinction training (average of 3 sessions preceding the reinstatement test, n = 7) and following bi-lateral NAc shell infusion of vehicle or DL-TBOA. DL-TBOA was microinfused ten minutes prior to the priming injection of cocaine (10 mg/kg, i.p.). No significant differences were found.

We next tested whether attenuating effect of DL-TBOA on maintenance of cocaine seeking is maintained following long withdrawal combined with extinction training. All animals received a microinfusion of vehicle or DL-TBOA (5 mM) into the NAc shell and then were administered a priming injection of cocaine (10 mg/kg i.p.). Although cocaine reinstated drug-seeking behavior in both vehicle and DL-TBOA treated animals, no differences in active lever (paired t-test, t_(6)_ = 0.72, p = 0.5; Fig 3A) or inactive lever (paired t-test, t_(6)_ = 1.72, p = 0.14; Fig 3B) presses were found between the two groups. We conclude that broad blockade of EAAT-mediated glutamate re-uptake does not affect cocaine-primed reinstatement of cocaine seeking.

### Experiment 3: DL-TBOA has no effect on the maintenance of sucrose self-administration

**Fig 4:**
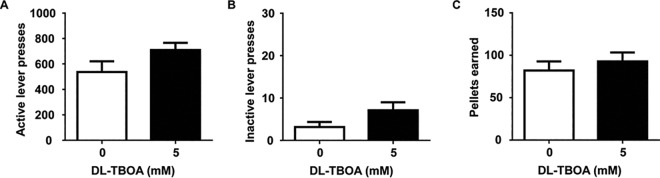
Microinjection of DL-TBOA did not affect sucrose self-administration. Total numbers (mean±SEM) of active lever responses (A), inactive lever responses (B), and sucrose pellets earned (C) were not different between animals that received a bilateral NAc shell microinfusion of vehicle and DL-TBOA, ten minutes prior to a 2 hour sucrose self-administration session (n = 6).

We evaluated whether DL-TBOA suppression of cocaine self-administration following short withdrawal can be extended to self-administration of naturally rewarding sucrose. Our results indicated that DL-TBOA had no effect on sucrose self-administration. The same dose of DL-TBOA (5mM) that significantly attenuated cocaine seeking had no effect on number of active lever presses for sucrose (t_(5)_ = 1.52, p = 0.19; Fig 4A) or total sucrose pellets earned (t_(5)_ = 0.85, p = 0.43; Fig 4C). Inactive lever presses were also not affected by DL-TBOA treatment (t_(5)_ = 1.88, p = 0.12; Fig 4B**).** Therefore, broad blockade of EAAT-mediated glutamate reuptake selectively attenuates self-administration of cocaine, but not sucrose, reward during early withdrawal.

### Experiment 4: DL-TBOA transiently increases locomotor activity

**Fig 5:**
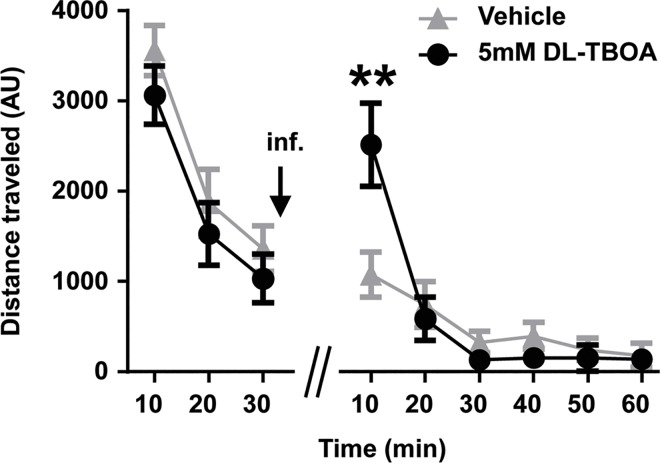
DL-TBOA transiently increased locomotor activity. Two groups of naïve animals habituated to a locomotor chamber during a 30-minute session with no difference between groups. Bilateral microinfusion (inf.) of vehicle or DL-TBOA into the NAc shell potentiated locomotor activity in the DL-TBOA group during the first ten minutes of the test session (Bonferroni, **p<0.01; n = 5). AU, arbitrary units.

We next examined whether the DL-TBOA-induced decrease in cocaine self-administration could be explained by general locomotor effects of DL-TBOA. Two groups of naïve animals were placed in locomotor chambers for a 30-minute habituation session following which one group received microinjections of DL-TBOA (5 mM), while the other received microinjections of vehicle into the NAc shell bilaterally and the locomotor activity was evaluated again for the next 60 minutes. During the habituation period, two-way repeated measures ANOVA revealed no significant effect of drug treatment, only significant main effect of time (F_(2,16)_ = 58.83, p<0.01; Fig 5). Post-hoc comparisons indicated a significantly higher basal locomotion at 10 min as compared to 20 and 30 min, consistent with a habituation effect. Following microinfusions, two-way repeated measures ANOVA revealed a significant main effect of time (F_(5,40)_ = 22.04, p<0.01) and time × treatment interaction (F_(5,40)_ = 5.80, p<0.01). No significant main effect of treatment was observed. However, post-hoc tests indicated that DL-TBOA transiently increased locomotor activity during the first 10 min post-injection as compared to vehicle treatment (Fig 5). We conclude that general locomotor effects of DL-TBOA do not account for attenuation of cocaine seeking behavior following short withdrawal from cocaine self-administration.

## Discussion

In this study, we examined the role of EAAT-mediated glutamate reuptake on subsequent cocaine seeking. We found that bilateral infusion of a broad spectrum EAAT-antagonist, DL-TBOA, into the NAc shell attenuated maintenance of cocaine self-administration following a short period of withdrawal, but did not affect cocaine-primed reinstatement of cocaine seeking following long withdrawal combined with extinction training. The suppressive effect of DL-TBOA on cocaine self-administration did not generalize to self-administration of naturally rewarding sucrose and was not a result of general locomotor impairment. Together, our results indicate that behavioral responses to blockade of glutamate re-uptake by DL-TBOA vary depending on the history of withdrawal from cocaine self-administration.

The present study was designed as a follow-up to our earlier report that following 1–2 days of withdrawal from limited access cocaine self-administration, activity of glutamate re-uptake transporters in NAc shell was increased, likely due to increased expression of EAAT1 and EAAT3 protein [13]. We argued that such an increase helps offset the impact of increased extrasynaptic NMDA receptor signaling and could contribute to lower basal extracellular glutamate concentrations reported following cocaine exposure [1,7,9]. In this study, we expected that elevation of extracellular glutamate by blockade of EAAT-mediated re-uptake would facilitate cocaine-related behaviors, consistent with the converging evidence that glutamate exerts permissive effects on cocaine seeking (reviewed in [23]). Contrary to this expectation, we found that DL-TBOA, attenuated cocaine seeking following one day of withdrawal from self-administration. One possibility that could account for this observation is co-regulation of EAAT and xCT1 activity. For example, N-acetylcysteine, a compound originally thought to stimulate xCT activity also increases expression of EAAT2 [17], while ceftriaxone, used to increase EAAT2 expression also increases expression of xCT1 [11]. In our study, blockade of glutamate re-uptake by DL-TBOA could down-regulate xCT1 expression resulting in lower extracellular glutamate levels and attenuated self-administration of cocaine. This explanation seems unlikely for several reasons. First, we observe DL-TBOA effects shortly after its administration, in contrast to the behavioral effects of N-acetylcysteine and ceftriaxone, observed after hours or days of pre-treatment with these compounds [6,12,16]. The quick action of DL-TBOA argues against changes in xCT1 expression as a mechanism of attenuated cocaine self-administration. Second, regardless of the interaction between EAAT blockade and xCT1 expression, acute administration of DL-TBOA rapidly increases extracellular glutamate *in vivo* and *ex vivo* as reported by many groups including ours [13,24,25]. However, the relative magnitude of DL-TBOA-driven increase in extracellular glutamate following short and long withdrawal/extinction may play an important role. Chronic administration of cocaine is associated with decreased basal NAc glutamate levels after both short and long withdrawal, but acute cocaine exposure only increases extracellular glutamate after extended withdrawal/extinction [1,6,7,8,9,26]. An increase in glutamate following a priming injection of cocaine in our reinstatement experiments may have occluded the effects of increased glutamate triggered by microinjection of DL-TBOA. Alternatively, the effects of DL-TBOA in reinstatement may have been masked by the already down-regulated EAAT2 levels observed following extinction training [11].

It is possible that DL-TBOA-driven increase in extracellular glutamate stimulated pre-synaptic, type II metabotropic glutamate receptors (mGluR). Activation of type II mGluRs signaling has been shown to attenuate cocaine seeking and reinstatement in multiple paradigms, including those involving manipulations of glutamate re-uptake [11,27,28,29]. Type II mGluRs are sensitive to low levels of extracellular glutamate and are known to reduce synaptic release of glutamate and other neurotransmitters [30,31]. DL-TBOA may further accentuate such reduction in synaptic release by disrupting glial EAAT-mediated supply of glutamate to neurons via the glutamate/glutamine cycle, resulting in accumulation of glutamate at extrasynaptic sites [13].

We found that DL-TBOA did not affect self-administration of sucrose in early withdrawal. Sucrose self-administration was measured in food-deprived animals which could contribute to lack of effect relative to *ad libutum*-fed cocaine-experienced animals. However, our findings are consistent with a similar lack of effect on sucrose self-administration recently reported following up-regulation of EAAT2 transport by ceftriaxone [32]. At the molecular level, DL-TBOA blocks glutamate re-uptake regardless of the behavioral training to self-administer sucrose or cocaine and specificity of behavioral effects of DL-TBOA to cocaine may again depend on the relative magnitude of extracellular glutamate increase. While cocaine self-administration decreases extracellular glutamate concentration, training to self-administer food reward does not alter extracellular glutamate levels [8]. Therefore, blockade of EAAT-mediated reuptake following sucrose self-administration can be expected to produce a smaller net increase in glutamate concentration than will the blockade of EAATs following self-administration of cocaine. However, attributing the early withdrawal behavioral effects exclusively to glutamate availability is problematic given that multiple neuroadaptations, including those not related to glutamate, are likely operative at this stage (e.g. [33,34,35]). An additional complication to consider is sensitivity of cocaine-induced molecular neuroadaptations to methodological differences in cocaine access [4,36,37,38]. In our case, manipulating behavioral history of access to cocaine could alter responses to DL-TBOA, not unlike manipulating behavioral history of withdrawal is seen to do.

Broad inhibition of glutamate re-uptake by L-trans-pyrrolidine-2,4-dicarboxylic acid (PDC) has been shown to dose-dependently increase spontaneous locomotor activity when injected into the NAc core [39]. Similarly, we find that DL-TBOA potentiated spontaneous locomotor activity when administered into the NAc shell of cocaine-naïve animals. Locomotor effects of PDC are more long-lived than those of DL-TBOA and persist for up to one hour following microinjection [39]. This may be due to differences in antagonist pharmacology (unlike DL-TBOA, PDC can be transported by EAATs in lieu of glutamate) or NAc core vs. NAc shell differences. We interpret the locomotor-activating effects of DL-TBOA to indicate that attenuation of cocaine self-administration that we observed was not a result of general sedation. Nevertheless, it remains possible that cocaine self-administration could have switched the direction of DL-TBOA effects on locomotor activity.

In summary, our study showed that acute blockade of EAAT-mediated glutamate reuptake in the NAc shell attenuated cocaine seeking specifically during the maintenance, but not reinstatement, of cocaine self-administration. These results support the evolving view that cocaine-induced neuroadaptations develop dynamically and reflect behavioral history of exposure to cocaine [4,36,37,38]. Distinct EAATs are likely engaged at different intervals of withdrawal and extinction of cocaine seeking and interact with other glutamate signaling pathways. It may be important to examine the nature of such interactions in future studies.
